# Characteristics of the Intestinal Microbiota in Very Low Birth Weight Infants With Extrauterine Growth Restriction

**DOI:** 10.3389/fped.2019.00099

**Published:** 2019-03-26

**Authors:** Hongping Li, Zhijiang He, Di Gao, Yuanhong Lv, Queyun Zhou, Bin Xiao, Weimin Huang

**Affiliations:** ^1^Shenzhen Children's Hospital, Shenzhen, China; ^2^Shenzhen University General Hospital, Shenzhen, China

**Keywords:** extrauterine growth restriction, very low birth weight, gut microbiome, 16S rRNA sequencing, preterm infants, neonatal intensive care unit

## Abstract

**Objective:** Very low birth weight (VLBW) infants, which experience significant postnatal growth restriction at the time of discharge, are at high risk of later growth failure and long-term consequences. This study aims to characterize the structure of intestinal microbiome community in VLBW infants with extrauterine growth restriction (EUGR).

**Methods:** Twenty-three VLBW infants appropriate for gestational age (GA) hospitalized at the neonatal intensive care unit of the BaoAn Maternal and Child Care Hospital (Shenzhen, China) were enrolled in this study, which were divided into the growth restriction group (EUGR; *n* = 12) and the normal growth group (AGA; *n* = 11). Meconium and fecal samples at postnatal day 28 were collected respectively during hospitalization. Total bacterial DNA was extracted and sequenced using the Illumina MiSeq Sequencing System based on the V3–V4 hyper-variable regions of the 16S rRNA gene.

**Results:** The intestinal bacterial communities of preterm infants were dominated by the phylum *Proteobacteria*. Compared with the AGA group, the relative abundances of the genera *Aeromicrobium* and *Serratia* in meconium samples significantly decreased, whereas genera *Parabacteroides, Ruminococcus, Blautia*, and *Aeromonas* were more prevalent in the EUGR group. On postnatal day 28, the relative abundances of the genera *Parabacteroides, Bacteroides, Eubacterium, Granulicatella*, and *Salinivibrio* were significantly different between the two groups, where genus *Salinivibrio* decreased significantly in the EUGR samples. Among them, genus *Parabacteroides* was more abundant on both postnatal day 1 and 28. Further KEGG prediction analysis showed that there were many differences in functional genes and pathways between the two groups on postnatal day 28, but not on day 1, the majority of which were related to energy metabolism. And no statistical differences were observed in the clinical characteristics of infants.

**Conclusions:** Overall, these findings showed that a distinct gut microbiota profile presented in preterm infants with EUGR. The role of intestinal microbiome in the extrauterine growth of preterm infants during hospitalization should be further investigated.

## Introduction

Failure to achieve adequate extrauterine growth is common in very low birth weight (VLBW) preterm infants (<1,500 g at birth), which known as extrauterine growth restriction (EUGR) (1). EUGR is most frequently defined as preterm infants below the 10th percentile for weight increases between birth and hospital discharge (2–4). That secondary to suboptimal nutritional status during a critical third trimester of the *ex-utero* preterm infant, would result in later morbidity and risk of adult onset diseases (5). Accumulating evidence has shown that EUGR not only affects physical development during infancy and childhood (6), but also increases the risk of hypertension (7), metabolic syndrome (8), such as diabetes and obesity, and impaired neurodevelopment (9), including neurological and sensory impairment, delayed cognitive development, and poor school performance.

The extrauterine growth is influenced by a complex interaction of multiple factors (10). And one of the most important factors is the difficulty to establish adequate nutrition in preterm newborns (11). In order to minimize the interruption of growth and development of preterm infants, aggressive nutritional approach has been proposed, with nutrient supply given more rapidly than suggested (12, 13). Despite of the continuing improvements in preterm infant care and great efforts made to improve neonatal nutrition, many infants failed to receive adequate nutrient intake and thus develop EUGR (14, 15).

Metagenomic analysis of the infant microbiome suggests that intestinal microbiome may play essential roles in the nutrition absorption, which is known to participate in energy harvest from the diet and to modulate host energy storage and metabolism (16, 17). Impaired composition of the intestinal microbiome is associated with weight gain and metabolic disease in children (18), and this effect can persist in adulthood (19). However, the associations between gut microbiota composition and extrauterine growth in preterm infants remains poorly understood. The aim of this study was to investigate whether gut microbiota composition of VLBW infants with EUGR exhibits a distinct profile at birth, and whether the differences persist post-natally.

Here, we performed a nested case-control study using fecal samples collected from VLBW infants on day 1 and 28 after birth. High-throughput 16S rRNA gene sequencing and bioinformatics analysis were used to determine genomic differences relate to EUGR.

## Materials and Methods

### Study Participants

In this cohort, VLBW preterm infants who admitted to the neonatal intensive care unit (NICU) of BaoAn Maternal and Child Care Hospital from January to December 2016 were enrolled. Selection criteria were as follows: gestational age (GA) <32 weeks, and birth weight (BW) < 1500 g. Infants with congenital malformation, intrauterine growth retardation, immune dysfunction, or severe infectious diseases; those who underwent fasting for more than 3 days during hospitalization; those with mothers who used immunosuppressive agents during pregnancy; those with guardians disagreeing with or withdrawing from the study; or infants who were abandoned or died within 2 weeks were excluded. The following maternal and neonatal information was obtained from medical records: maternal age, duration of ruptured membranes, clinical chorioamnionitis, maternal hypertension, and diabetes; antenatal treatment with dexamethasone and magnesium sulfate; mode of delivery, GA, BW, postnatal age, gender, small for gestational age (SGA), singleton/multiple gestation, early-onset and late-onset sepsis, respiratory distress syndrome (RDS), patency of the ductus arteriosus (PDA), postnatal treatment with steroids, days of antibiotic treatment during the entire hospital stay, red blood cell transfusions, feeding type (exclusive maternal/donor breast milk, exclusive formula, and mixed), NEC (Bell stages II or III), feeding intolerance which defined as gastric residual volume more than 50% and abdominal distention or emesis of both, and the length of hospital stay. The body weight at discharge of each infant was compared with the 10th percentile of expected values. Infants with measured growth values at discharge of ≤ 10th percentile of the predicted value were assigned to the EUGR group (EUGR) and others were assigned to the normal growth group (AGA). The study protocol was approved by the medical ethics committee of BaoAn Maternal and Child Care Hospital. Written informed consent was obtained from the parents or guardians of the infants.

### Sequencing and Bioinformatics Analysis

Extraction of DNA was carried out as follows: First, fecal samples were collected using sterile swabs from the diapers of infants. Swabs were stored in 1 μL cell lysis solution immediately after collection and subsequently stored in −80°C freezer until further processing. DNA was extracted using a commercially available kit (QIAamp Fast DNA Stool Mini Kit; Qiagen, Valencia, CA, USA) according to the manufacturer's instructions.

DNA Sequencing was performed as follows: First, DNA concentration and quality were determined by Qubit and verified using agarose gel electrophoresis. Primers 5′-CCTACGGGNGGCWGCAG-3′ (forward) and 5′-GACTACHVGGGTATCTAATCC-3′ (reverse) were used to amplify the 16S rRNA V3–V4 variable regions. Equimolar amounts of purified PCR products were pooled and processed for sequencing. DNA sequencing was performed on an Illumina MiSeq instrument with barcoding using a sequence kit (version 3.0) for optimal pair-end sequence reads.

The data was first preprocessed with Trimmomatic (20). The specific processing rules were as follows: (1) bases with quality below 25 were removed from the end; (2) 50-bp sliding window, 1-bp step shift, windows with average quality <25 were also removed; and (3) sequences with a length below 50-bp were removed. Then FLASH (21) was used to splice high-quality paired end sequences and to remove sequences with fuzzy bases. According to the barcode, the sequences of every sample were split and then aligned to the *Homo Sapiens* genome to remove the polluting host sequences using Bowtie2 (22). After removal of chimeric sequences with UCHIME (23) and clustering of 16S rRNA gene sequences with UPARSE (24), sequences similarities of more than 97% were clustered into the same operational taxonomic units (OTUs). Substantial taxonomic ranks were assigned using the Ribosomal Database Project Naïve Bayesian Classifier (25) against the GreenGenes database (26) with an 80% value threshold. Pathway prediction was performed using Picrust (27). And the LDA Effect Size (LEfSe: Linear Discriminant Analysis Effect Size) algorithm was used to identify taxa with differentiating relative abundance (28). The threshold for the logarithmic LDA score was set at 2 for biomarker discovery.

### Data Analysis

Characteristics were analyzed using R. Continuous variables were reported as means ± standard deviations, and categorical data were presented as ratios or percentages. Unpaired *t*-tests were used to study differences in continuous variables and χ^2^ tests were used to analyze categorical variables. Difference with *P* < 0.05 was considered statistically significant.

## Results

### Demographic and Clinical Characteristics of Infants

In this cohort, 40 VLBW infants with a GA of <32 weeks were treated in the NICU of BaoAn Maternal and Child Care Hospital. Based on the exclusion criteria, 17 cases were excluded, and the remaining 23 cases were included in the final analysis. Based on the discharge weight, the 23 enrolled infants were divided into the EUGR and AGA group. There were no significant differences in any of the demographic and clinical characteristics between the two groups. The demographic and clinical data of the enrolled subjects were summarized in Table 1.

**Table 1:** Demographic and clinical characteristics of the two groups.

**Characteristic**	***N* = 23**	**EUGR = 12**	**AGA = 11**	***P*-value**
Gestational age, weeks (mean, SD)	28.9 (1.8)	29.2 (1.9)	28.5 (1.7)	0.31
Maternal age, years (mean, SD)	28.7 (5.1)	28.6 (5.7)	28.7 (4.5)	0.95
Birth weight, grams (mean, SD)	1,187 (212)	1,187 (217)	1,188 (216)	0.99
Male	18 (78.3%)	9 (75%)	9 (81.8%)	1
Antenatal medications				
Antibiotic	9 (39.1%)	5 (41.7%)	4 (36.4%)	1
Magnesium sulfate	18 (78.3%)	7 (58.3%)	11 (100%)	0.06
Dexamethasone	21 (91.3%)	11 (91.7%)	10 (90.9%)	1
Vaginal birth	12 (52.2%)	7 (58.3%)	5 (45.5%)	0.84
Multiple birth	7 (30.4%)	4 (33.3%)	3 (27.3%)	1
Chorioamnionitis	13 (56.5%)	5 (41.7%)	8 (72.7%)	0.28
Small for gestational age	0 (0%)	0 (0%)	0 (0%)	1
Amino acid supplement	22 (95.7%)	12 (100%)	10 (90.9%)	0.96
Respiratory distress syndrome	10 (43.5%)	4 (33.3%)	6 (54.5%)	0.55
Patent ductus arteriosus	8 (34.8%)	6 (50%)	2 (18.2%)	0.25
Postnatal age, weeks (mean, SD)	37.2 (2.0)	37.6 (2.1)	36.7 (1.9)	0.28
Postnatal treatment with steroids	3 (13.0%)	1 (8.3%)	2 (18.2%)	0.94
Bronchopulmonory dysplasia	13 (56.5%)	6 (50%)	7 (63.6%)	0.81
Treated retinopathy of prematurity	5 (21.7%)	3 (25%)	2 (18.2%)	1
Necrotizing enterocolitis	1 (4.3%)	1 (8.3%)	0 (0%)	1
Surgical necrotizing enterocolitis	1 (4.3%)	1 (8.3%)	0 (0%)	1
Postnatal antibiotics exposure in the first week	18 (78.3%)	10 (83.3%)	8 (72.7%)	0.91
Postnatal antibiotics exposure after the first week	14 (60.9%)	7 (58.3%)	7 (63.6%)	1
Days on antibiotics during hospitalization (mean, SD)	14.5 (10.5)	16.3 (13.5)	12.4 (6.0)	0.44
Early onset infection	14 (60.9%)	8 (66.7%)	6 (54.5%)	0.87
Late onset infection	10 (43.5%)	6 (50%)	4 (36.4%)	0.81
Packed red blood cell transfusion	20 (87.0%)	9 (75%)	11 (100%)	0.25
Feeding type				0.24
Maternal breast milk only	5 (21.7%)	1 (8.3%)	4 (36.4%)	
Formula only	8 (34.8%)	5 (41.7%)	3 (27.3%)	
Mixed feeding types	10 (43.5%)	6 (50%)	4 (36.4%)	
Feeding intolerance in post-natal 2 weeks	11 (47.8%)	7 (58.3%)	4 (36.4%)	0.52
Length of stay, days (mean, SD)	58.4 (21.6)	58.9 (24.1)	57.7 (19.7)	0.90
Postmentrual age, weeks (mean, SD)	37.3 (1.8)	37.8 (1.9)	36.7 (1.6)	0.16
z-score for weight at discharge	−1.5 (0.65)	−2.0 (0.5)	−1.0 (0.1)	<0.01

To profile the gut microbiota community structure, the V3–V4 regions of the bacterial 16S rRNA gene were sequenced. In total, 44 fecal samples were collected from the enrolled infants on postnatal day 1 and 28, and 3,767,747 reads were obtained, each sample has an average of 85,631 reads. The collected fecal specimens on day 1 and 28 in the EUGR group were labeled: EUGRd1 and EUGRd28, respectively, the counterparts in the AGA group were designated AGAd1 and AGAd28, respectively.

### Overall Microbiota Structures of Intestinal Microbiota

**Figure 1:**
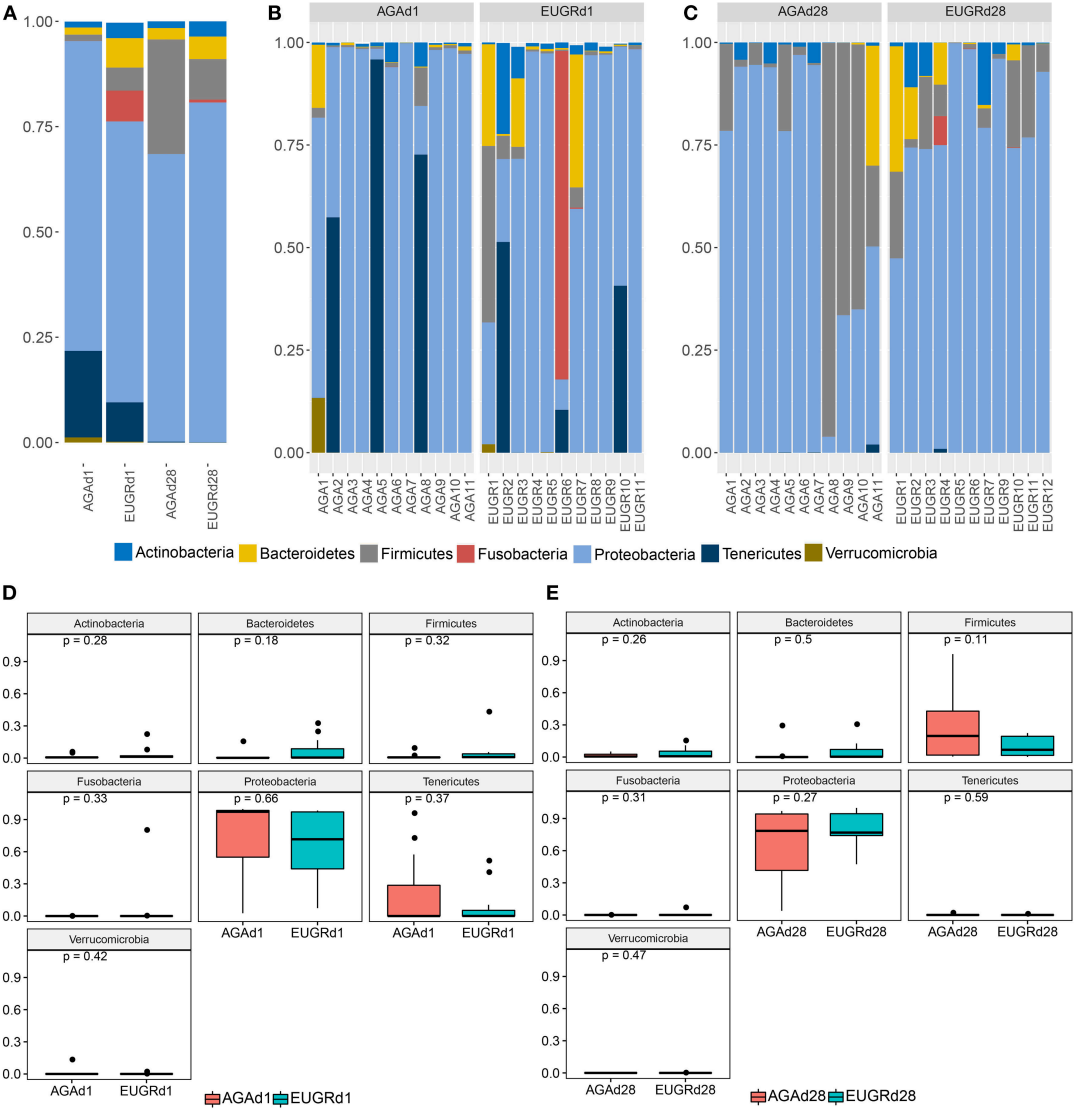
Overall structures of intestinal microbiota at the phylum level. **(A)** Average microbiome community of group EUGR and AGA. **(B)** Microbiome community structure of each sample at d1. **(C)** Microbiome community structure of each sample at d28. **(D)** Differences of phylum between EUGRd1 and AGAd1 group. **(E)** Differences of phylum between EUGRd28 and AGAd28 group.

Next, we studied the intestinal microbiota structure of infants with or without EUGR. The main phyla in the EUGRd1 and AGAd1 groups were *Actinobacteria, Bacteroidetes, Firmicutes, Fusobacteria, Proteobacteria, Tenericutes*, and *Verrucomicrobia* (Figure 1B). Compared with the AGAd1 group, the proportions of *Proteobacteria* (66.71 vs. 73.57%), *Tenericutes* (9.33 vs. 20.53%), and *Verrucomicrobia* (0.21 vs. 1.22%) decreased, and the proportions of *Actinobacteria* (3.63 vs. 1.38%), *Bacteroidetes* (6.99 vs. 1.69%), *Firmicutes* (5.47 vs. 1.52%), and *Fusobacteria* (7.31 vs. 0.01%) increased in the EUGRd1 group. Furthermore, the main phyla in the EUGRd28 and AGAd28 groups were *Actinobacteria, Bacteroidetes, Firmicutes*, and *Proteobacteria* (Figure 1C). The proportions of *Actinobacteria* (3.60 vs. 1.58%), *Bacteroidetes* (5.35 vs. 2.70%), and *Proteobacteria* (80.64 vs. 68.30%) increased in the EUGRd28 group, whereas that of *Firmicutes* (9.62 vs. 27.20%) decreased compared with the AGAd28 group. The trends of changes were observed without significant differences on both postnatal day 1 and 28 (Figures 1D,E). Additionally, at day 28, the phylum *Tenericutes* decreased, whereas the phyla *Proteobacteria* and *Firmicutes* increased compared with those on day 1 both in the EUGR and AGA groups (Figure 1A). And the class *Gamaproteobacteria* dominated the phylum *Proteobacteria*, the average relative abundance was 70.4% in AGAd1, 67.9% in AGAd28, 60.8% in EUGRd1, 79.2% in EUGRd28.

**Figure 2:**
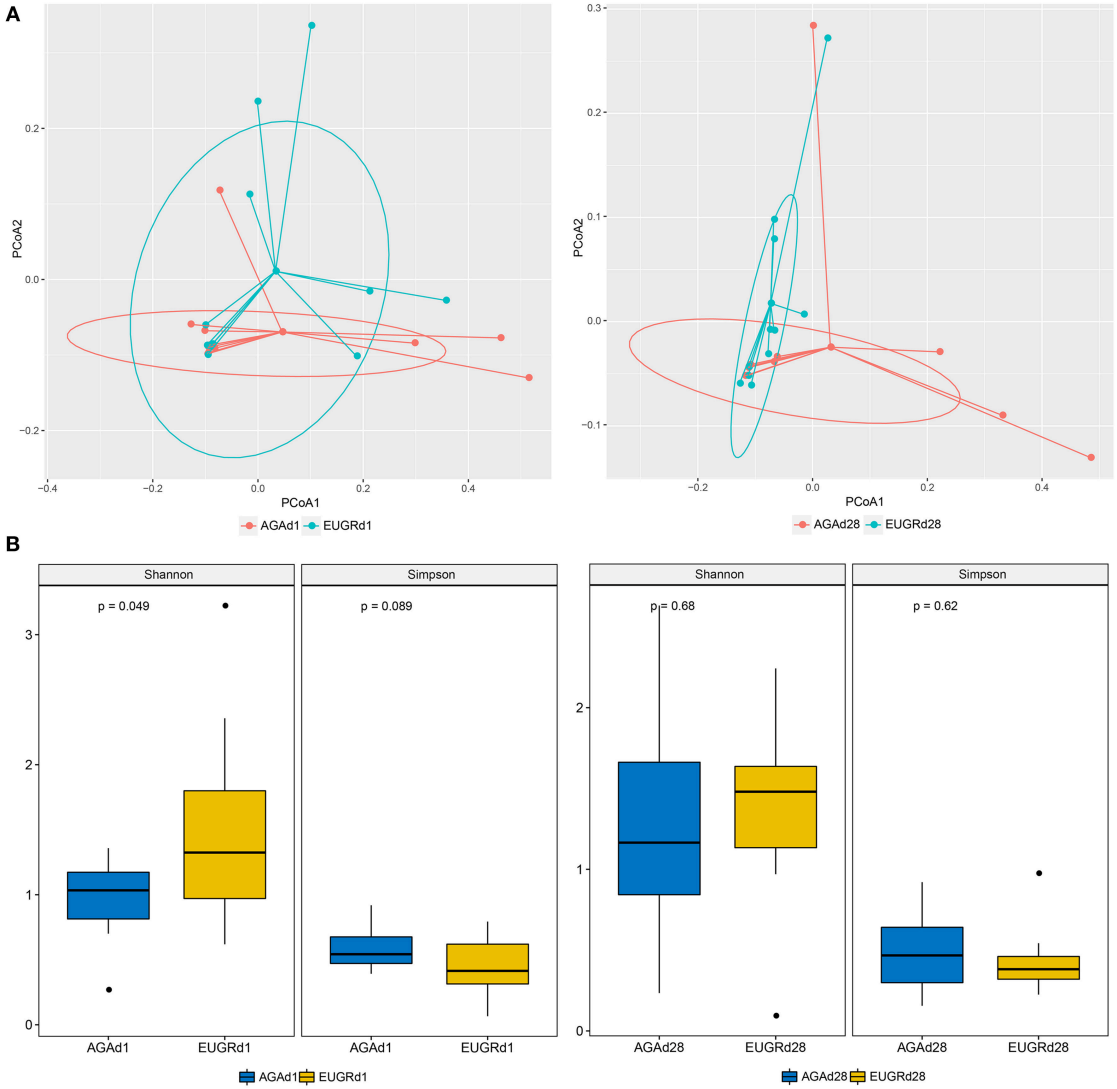
Principal coordinate analysis and alpha diversity plots in intestinal microbiota. **(A)** Weighted UniFrac PCoA plot based on OTU abundance. Each point represents the intestinal microbiota of a newborn in the EUGR group (red) and AGA group (green). **(B)** Comparison of the microbiome biodiversity between infants with EUGR and normal growth, the Shannon index, Simpson index were shown as estimators.

To compare the overall intestinal microbiota structures in infants with postnatal growth failure and normal infants, principal coordinate analysis (PCoA) was implemented based on the OUT level. On both day 1 and 28, the results of PCoA showed difference in bacterial structure of gut microbiome between the EUGR and AGA groups, and many overlap subjects existed (Figure 2A). The average weight unifrac distance value of all subjects decreased from d1 (0.37) to d28 (0.26). While the distance decreased from 0.35 to 0.17 in the EUGR group, it also decreased from 0.33 to 0.29 in the AGA group. The microbiome community presented a higher similarity in infants with EUGR compared to AGA group at d28. Moreover, the average Shannon and Simpson values were 0.97 ± 0.31, 0.58 ± 0.16 in AGAd1 group (mean ± SD), 1.50 ± 0.78 and 0.44 ± 0.22 in EUGRd1, 1.25 ± 0.67 and 0.48 ± 0.24 in AGAd28, 1.36 ± 0.55 and 0.43 ± 0.21 in EUGRd28. There was a higher value of Shannon index presented in EUGRd1 compared to AGAd1 with *P* < 0.05 (Figure 2B).

### Genus-based Comparisons of Intestinal Microbiota Between EUGR and AGA Groups

**Figure 3:**
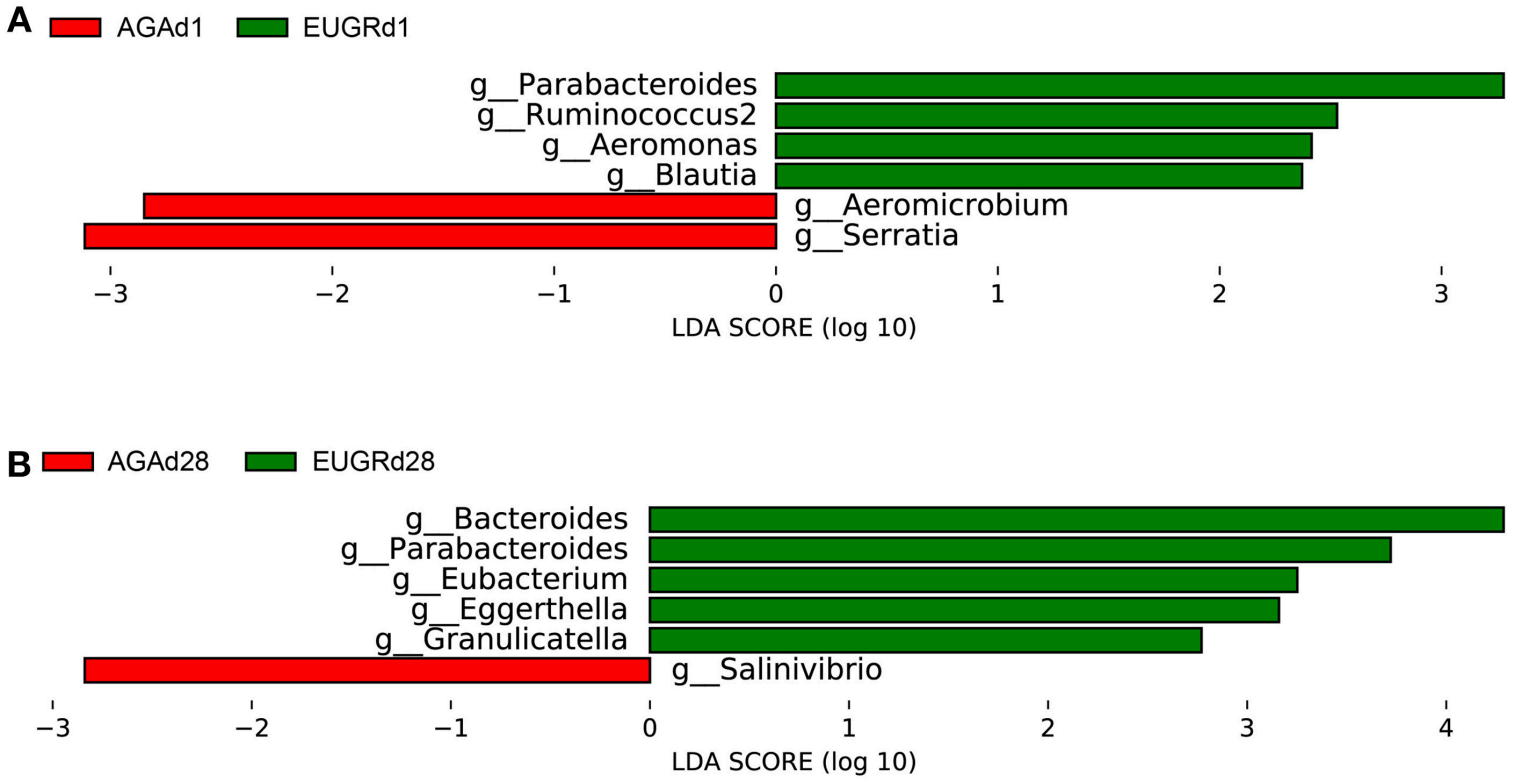
Histogram of the LDA scored for differentially abundant genera between EUGR and AGA groups (red: AGA; green: EUGR). LDA scores were calculated by LDA effect size using linear discriminant analysis. **(A)** was d1, and **(B)** was d28.

Next, the LEfSe tool (28) was used to analyze bacterial communities in fecal samples and to detect potential significant differences in relative abundances between the EUGR infants and normal growth infants. Significant variations in the communities of the intestinal microbiota were observed at the genus level. Figure 3 included a list of genera that significantly shifted between the EUGR and AGA groups on day 1 and 28. There were significant differences in 6 genera between the EUGRd1 and AGAd1 groups. *Parabacteroides, Ruminococcus2, Blautia*, and *Aeromonas* were relatively more abundant, whereas *Aeromicrobium* and *Serratia* in the EUGRd1 group exhibited a relatively lower abundance than that in the AGAd1 group (Figure 3A). In addition, the results showed that genera *Bacteroides, Parabacteroides, Eubacterium, Granulicatella*, and *Eggerthella* significantly increased in the EUGRd28 group compared with that in the AGAd28 group, whereas genus *Salinvibrio* showed a significant decrease in abundance (Figure 3B). Among the results, genus *Parabacteroides* was the only bacterium that was highly abundant in the growth retardation group on day 1 and 28 both. Collectively, these observations suggested that the microbial composition of the gut significantly differed between infants with and without EUGR according to the relative abundance of sequences.

### Functional of Metabolism in the Gut Microbiome

**Figure 4:**
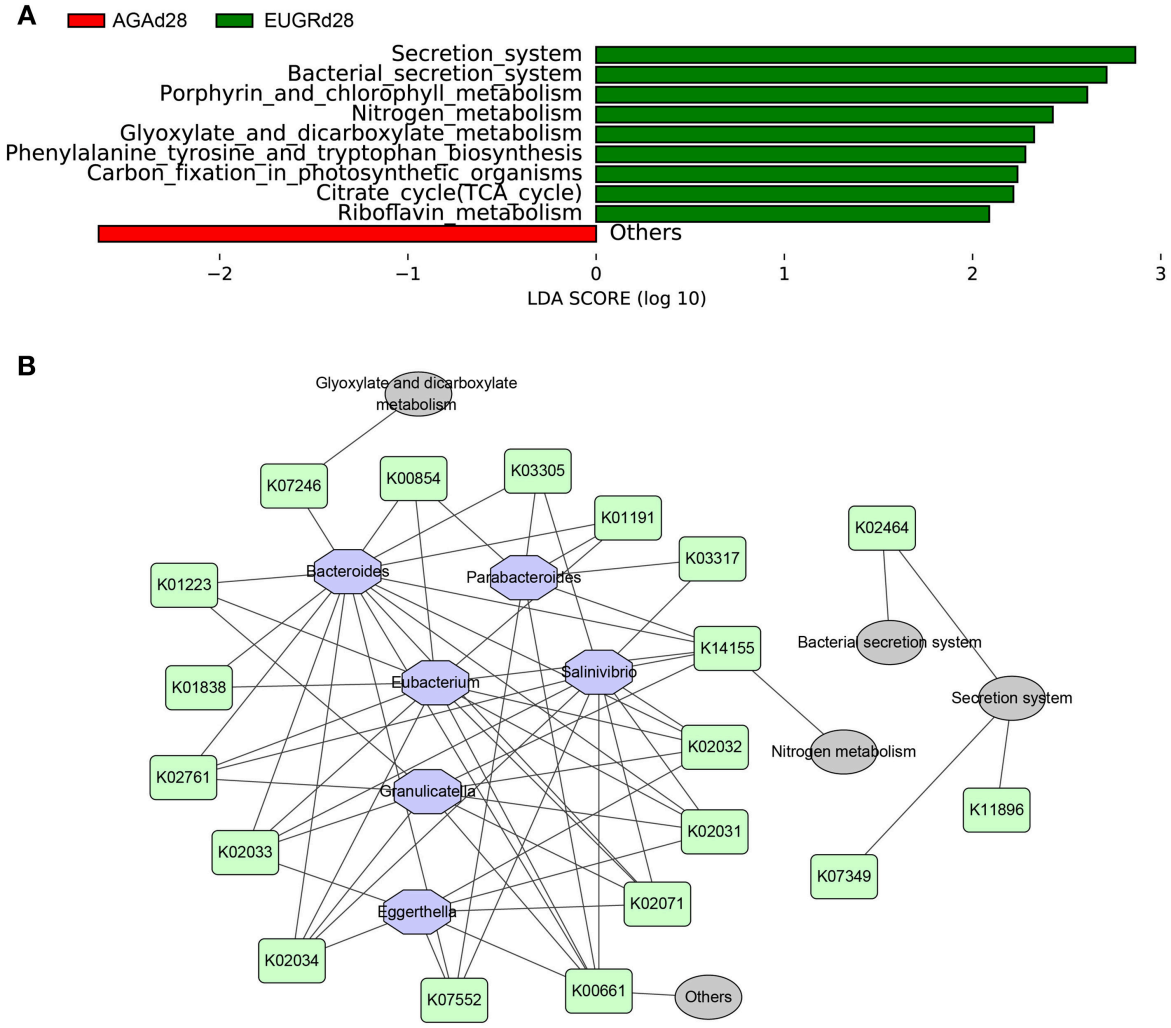
Differently abundant pathways between EUGR and AGA groups and its relationship with varying abundances of genera. **(A)** Functional properties that differed significantly between EUGRd28 and AGAd28 groups. **(B)** Relationships among bacteria, functional genes, and metabolic pathways.

To investigate the changes in microbiome metabolites induced by changes in the microbiome abundance, functional prediction and pathway analyses of bacterial DNA in the two groups were performed using gene sets of the Kyoto Encyclopedia of Genes and Genomes (KEGG). There were no differences in functional genes or pathways between the EUGRd1 and AGAd1 groups. In contrast, significant differences in several functional genes and pathways were found between the EUGRd28 and AGAd28 groups (Figure 4A). The gut microbiome of infants with EUGR exhibited more abundant involvement of the secretion system, citrate cycle, nitrogen metabolism, glyoxylate and dicarboxylate metabolism, phenylalanine tyrosine and tryptophan biosynthesis, porphyrin and chlorophyll metabolism, and riboflavin metabolism. In order to elucidate the relationships between the different taxa, functional genes, and pathways, a network diagram was designed (Figure 4B).

## Discussion

Achieving good postnatal growth in preterm infants is still a major clinical challenge (1). Recent studies on metabolism and metabolic diseases suggest that gut microbiome directly impacts growth and development of preterm infants (29, 30). And maturation of the gut microbiota in early life is linked to physiological development with long-term influences on infant health (31). In this study, 16S rRNA sequencing was used to detect differences in the gut microbiome between VLBW infants with EUGR and normal growth. PCoA was applied to visualize the dissimilarity of bacterial populations between EUGR and AGA group, and differences were presented both on postnatal day 1 and 28, indicating that the differences persist partly after birth. In accordance with the previous study (32), similarity of the microbiome community in preterm infants increased over time in this study. And smaller average weight unifrac distance value was observed in the EUGRd28 compared to AGAd28, indicating infants with EUGR during hospitalization were less dissimilar. The main phyla in VLBW infants were *Actinobacteria, Bacteroidetes, Firmicutes*, and *Proteobacteria*. Among them, *Proteobacteira* was found to be the most abundant phylum, accounting for more than 68% of total reads, regardless of the presence of EUGR. The abundance of phylum *Proteobacteria* increased over time, and the class *Gammaproteobacteria* dominated the *Proteobacteria*, comprising more than 60% of total sequences. This result was consistent with a previous study (33). Class *Gammaproteobacteria* contained families *Enterobacteriaceae, Vibrionaceae*, and *Pseudomonadaceae* that might be associated with adverse outcomes, including necrotizing enterocolitis, late-onset sepsis, and development delay (34–36). As recent study demonstrated that infections, subclinical pathogen carriage of gut microbial communities underlies malnutrition in children (17). Lower taxonomic levels, larger cohort, and experimental studies should be taken into account in further study to confirm the identified contributors and to elucidate the mechanism.

Furthermore, LDA analysis demonstrated that distinct profile of intestinal microbiome existed in the preterm infants with failure growth and normal growth at postnatal day 1 and 28. Notably, the genus *Parabacteroides* was the only taxa highly abundant in the EUGR group at both time points. Genus *Parabacteroides* is a gram-negative, anaerobic, non-spore-forming genus, some species of which produce high quantities of acetic acid and propionic acid (37). Additionally, acetic acid and propionic acid are short-chain fatty acids that can be absorbed by intestinal epithelial cells (IECs) and eventually enter the blood circulation, affecting the storage of sugar in muscle, liver, and fat. Moreover, acetic acid also can reach the brain, causing loss of appetite, and resulting in reduced food intake (38). Thus, excessive colonization of genus *Parabacteroides* in the intestinal tract of VLBW infants might relate to weight gain. But it was still unclear the mechanism and effect of genus *Parabacteroides* on weight gain. Larger cohort and experimental studies toward a better understanding of its effect should be conducted in the next stage.

Some studies have demonstrated that genus *Serratia* can secrete hemolysin, a pore-forming toxin that causes transformation of the intestinal epithelium in insects and mammals (39, 40). In response to hemolysin, enterocytes extrude most of their apical cytoplasm, including damaged organelles, such as mitochondria, resulting in an apparent thinning of the epithelium without gut leakage or cell lysis. Surprisingly, the affected IECs grow back to their initial thickness after a few hours (41). In our study, although the relative abundance of genus *Serratia* was higher in the AGAd1 group than that in the EUGRd1 group, its influences on IECs renewal and enteral nutrition absorption should be investigated and validated in further studies.

Undernutrition was certainly influenced by metabolic impact of dysbiotic commensal of gut microbiota (42). Functional analysis with KEGG database showed that samples from the EUGRd28 and AGAd28 groups contained many different functional genes, whereas those from the EUGRd1 and AGAd1 groups did not. In addition, metabolic pathway analysis revealed that genes related to the bacterial secretion system and environmental information processing-related metabolic pathways, including riboflavin metabolism, citrate cycle, carbon fixation in photosynthetic organisms, phenylalanine tyrosine and tryptophan biosynthesis metabolism, glyoxylate and dicarboxylate metabolism, nitrogen metabolism, and porphyrin and chlorophyll metabolism, increased in EUGRd28. Notably, most of these pathways were linked to energy metabolism.

EUGR remains a frequent and universal problem in preterm infants with VLBW in neonatal intensive care units, and it caused by multi-factors. Previous study suggested that multiple clinical variables, maternal hypertension, SGA, PDA, RDS and hospitalization time, and so on, influenced growth restriction (43). Such SGA infants were discharged with greater growth restriction (44). Moreover, evidences in premature infants suggested that gut microbiota development was associated with GA, but was also shaped by the restricted environment in the neonatal intensive care unit, infant nutrition, and common clinical practices in neonatal care (45–47). In this study, the perinatal and neonatal clinical characteristics presented no significant differences between the EUGR and AGA group.

The meconium was previously thought to be sterile, yet numerous studies have since reported a complex meconium microbiota signature (48, 49). The same phenomenon was observed in this study, but also there were many significant differences in the meconium samples between EUGRd1 and AGAd1 group. Fetal microbiome in the meconium was similar to the placental and amniotic microbiota (50), and *in utero* ingestion of microbes present in the amniotic fluid partly leaded to the bacterial colonization of the fetal gut. The difference in the meconium microbiome suggested that some signatures may exist in the maternal status during pregnancy. On the other side, the acquisition of first gut microbial colonizers was affected by multiple maternal factors, such as mode of delivery, maternal intrapartum antibiotic use, clinical chorioamnionitis, and so on (17). The differences noted on meconium samples might reasonably be attributed to differences of these factors between the two groups. However, the small sample size was insufficient to either validate or reject these possible explanations in this study. A large sample size must be taken into account in further studies.

However, several potential limitations should be taken into consideration. First, the sample size was small and sampling time was limited. In addition, due to that all the participants included in this study were all recruited in the same hospital, hence potential regional differences in placental microbiota was not assessed. A multicenter clinical study to fully investigate and compare the intestinal microbiota of subjects with EUGR among different regions should be designed. Further, since there were no data on the feeds differences between the two groups, the effect of different feeds on the difference in microbiome was not comprehensively understood. Moreover, BW is a continuous variable but treated as a dichotomous one in this study, which presented a little arbitrariness. As z-score for weight is one way of quantifying extent of EUGR, the relationships between the gut microbiome community and z-score need to be investigated in the next study.

In conclusion, we found that infants with EUGR showed distinct intestinal microbiota profiles at postnatal day 1 and 28. These findings provided insights into the potential relationship between intestinal microbiota and EUGR, but it was yet unclear whether this microbial presence is a cause or effect of impaired growth. As the high prevalence of infants with EUGR, a better understanding of the connections between the intestinal microbiome and the development of EUGR is important for improving infant health. Thus, high-quality, large-scale clinical studies and further animal and preclinical experiments are needed to clarify the underlying mechanisms and establish methods for the prevention and treatment of EUGR.

## Author Contributions

WH and HL conceived of the presented idea and planned the experiments. ZH, DG, YL, and QZ carried out the experiments. HL and BX designed the computational framework and analyzed the data. HL and ZH wrote the manuscript. All authors discussed the results and contributed to the final manuscript.

### Conflict of Interest Statement

The authors declare that the research was conducted in the absence of any commercial or financial relationships that could be construed as a potential conflict of interest.
